# Supplementary material for: Antibacterial, Antibiofilm, and Antioxidant Activity of 15 Different Plant-Based Natural Compounds in Comparison with Ciprofloxacin and Gentamicin
Source: Antibiotics (Basel). 2022 Aug 12;11(8):1099. doi: 10.3390/antibiotics11081099 (PMC9404727; doi:10.3390/antibiotics11081099)
Supplement: Supplementary file 1 [file antibiotics-11-01099-s001.zip › antibiotics-1848715-supplementary.pdf]

Supplementary

Antibacterial, Antibiofilm, and Antioxidant Activity of 15 different Plant-based Natural compounds in Comparison with Ciprofloxacin and Gentamicin.

Ali Pormohammad[1,2], Dave Hansen[1], Raymond J. Turner[1]*

1. Department of Biological Sciences, Faculty of Science, University of Calgary, Calgary, Alberta, Canada.
2. CCrest laboratories Inc, Montreal, Quebec, Canada.

***** Corresponding author: turnerr@ucalgary.ca
Mail address: 447-Biological Sciences Building, 2500 University Dr. NW, Calgary, Alberta T2N 1N4, Canada; Tel.: +1-403-220-4308

1. Supplementary Materials and methods

1.1. Systematic review for selecting highly effective plant-based natural compounds (PBCs) as antibacterial agents

We conducted a systematic review of available publications to generate a list of potentially highly effective antibacterial plant-based natural compounds (PBCs). The systematic review was performed according to the Preferred Reporting Items for Systematic Reviews and Meta-Analyses statement (PRISMA) guidelines (1). We searched all pieces of literature from January 1, 2000, to September 1, 2021, from Scopus, Embase, Medline (via PubMed), and Web of Science. Search medical subject headings (MeSH) terms used were: “natural compounds”, “natural product”, “herbal extract”, “herbal medicine”, “antibacterial”, “antimicrobial”, “Cannabidiol”, “CBD”, “Cannabis sativa”, “THC”, “tetrahydrocannabinol”, “Cinnamaldehyde”, “Tea Tree Oil”, “Nerolidol”, “Carvacrol”, “*0*-coumaric acid”, “Thymol”, “Thymic acid “, “Canada Balsam”, “resveratrol”, “curcumin” and their synonyms. Moreover, we searched for unpublished and grey literature with Google scholar. We also assessed the references of included identified articles and related reviews to find additional relevant studies. After screening titles and abstracts, the full text of potentially eligible records was examined and retrieved.

Inclusion and exclusion criteria studies had to fulfil the pre-determined criteria to be eligible for inclusion in our systematic review. To facilitate the comparison of our results with those in other publications, studies with minimum inhibitory concentration (MIC) data were included in our systematic review. Studies that reported non-quantitative antibacterial susceptibility with the zone of inhibition diameter or any other method other than MIC, were excluded from this study. The following items were extracted from each article: first author, publication date, bacteria name, MIC, and important relevant comments from each study. Quality assessments of included studies were performed according to the critical appraisal checklist recommended by the Joanna Briggs Institute (2). The checklist is composed of nine questions, the 'Yes' answer to each question received one point. Thus, the final scores for each study could range from 0 to 9.

1.2. Bacterial strains and culture media

Based on the company's instructions, bacterial strains were stored at − 70 °C in Micro-bank vials (Richmond Hill, Ontario, Canada). Six indicator strains were used for all experiments; including *Pseudomonas aeruginosa* ATCC 27853, *Klebsiella pneumoniae* ATCC 11296, *Staphylococcus aureus* ATCC 25923, *Escherichia. coli* ATCC 25922, *Proteus mirabilis* MMX 6442, and *Acinetobacter baumannii* CDC strain AR Bank # 0033 (3). Mueller-Hinton Broth (MHB, BD Bacto, Oxoid, Basingstoke, UK Cat# X243B) was used as the growth medium for susceptibility testing media in this study (4, 5).

1.3.Antibiotics and Plant-based natural compounds (PBCs)

We generated a list of eleven of the most potent antibacterial PBCs by the systematic review of available publications. These eleven PBCs are Cannabidiol (CBD) (SIGMA, MO, USA, LOT#SLCC9048), (-)-11-nor-9-carboxy-Δ9-tetrahydrocannabinol (THC) (SIGMA, MO, USA, LOT#FE05081905), Cinnamaldehyde (Fisher Scientific, Ottawa, Canada, LOT #5018R23W, >98%), Tea Tree Oil (Newconatural, Australia, LOT 80038607, >98%), Nerolidol (TCI, Portland, USA LOT #IKUMH-BE, >97%), Carvacrol (TCI, Portland, USA LOT #NDFYD-GT, >98%), *0*-coumaric acid (SIGMA, MO, Germany, LOT#106H0966), Thymol (Fisher Scientific, Ottawa, Canada, LOT #W12E020, >98%), Resveratrol (TCI, Portland, USA LOT #QWDXI-RF, >99%), Curcumin (Alfa Aesar, Ontario, Canada LOT #1022628, >95%), and Canada Balsam (SIGMA, MO, USA, LOT#125H2518). The two most common antibiotics, gentamicin (Amresco, Solon, Ohio, USA LOT #339C337) and ciprofloxacin (6) (SIGMA, MO, Germany, LOT#146C0896) were used as controls to provide a comparison of the antibacterial properties of the PBCs.

1.4.Minimum Inhibitory Concentration (MIC) Assay

All planktonic and biofilm susceptibility testing were carried out using the Calgary biofilm device; commercially available as the MBEC physiology and genetics assay [Innovotech Inc., Edmonton, Alberta, Canada]), as originally developed and described bythe Calgary Biofilm Research Group (7). This device is a peg lid fitted into a 96 well microtiter plate. Serial dilutions of each PBC, with a dilution factor of two, were prepared; the first column served as a negative control (Media, 0 mM PBCs and no bacteria) and the last column served as a positive control

(Media and bacteria, 0 mM PBCs with bacteria). Briefly, − 70 °C stored bacteria were sub-cultured two times overnight (O/N) at 37 °C on MHA plates to obtain a pure single colony. 75 µL of 1.0 X 10^6 CFU/ml inoculum of bacteria were then added to each well and the plate was incubated for 24 hours at 37 °C in a microplate shaker at 150 rpm (5). The well with the highest dilution, but which did not show evidence of bacterial growth, was considered as the minimal inhibitory concentration (MIC). Bacterial growth was determined by reading the optical density at 600 nm (OD600), using a Thermomax microtiter plate reader with Softmax Pro data analysis software (Molecular Devices, Sunnyvale, CA). Due to unclear MIC for some of the PBCs that gave high solution turbidity, colony-forming units (CFU) were obtained for the determination of the exact MIC.

1.5.Minimum Bactericidal Concentration (MBC) Assay

At the end of the MIC determination experiment, 10 µL of each MIC well were transferred in 140 µL of the same fresh media in a new 96 plate and incubated 24 hours at 37◦C in a microplate shaker at 150 rpm. MBC was determined by reading the optical density at 600 nm (OD600) of the recovery plates using a Thermomax microtiter plate reader with Softmax Pro data analysis software (Molecular Devices, Sunnyvale, CA).

1.6.Prevention of biofilm

All biofilm experiments were carried out using a Calgary biofilm device. Briefly, -70 °C stored bacteria were sub-cultured on MHA at 37 ◦C overnight (O/N) to obtain a pure single colony. 75 µL of the desired concentration of PBCs was added to 96 wells, 75 µL of bacteria (1.0 X 10^6 CFU/ml) added in each well, finally the polystyrene CBD pegged lid was placed into the 96 wells and incubated 48 hours at 37°C in a microplate shaker incubator at 150 rpm. The CBD lids were removed from the media and the adhered biomass was rinsed two times with distilled water. The extent of the biofilm biomass was determined using a crystal violet assay (8), which allowed the minimum biofilm inhibitory concentration (MBIC) to be determined. The well with the highest dilution of PBCs, but which had no bacterial biofilm and zero OD600 absorption, were considered MBIC. Results from at least three separate biological replicates were reported (4, 9).

1.7. Cannabis sativa oil Extraction

Preliminary results showed promising antibacterial and anti-biofilm features for high purity CBD and THC. Therefore, the whole plant of two different cultivars of *Cannabis sativa* (sample #98 and #112) was extracted with food-grade extra virgin olive oil or canola oil to compare the results with ultra-pure components. In a simple extraction method, ground plant flower heads were added to each oil in the desired concentration and stirred for four hours at 80 °C. The extraction was then used for the same antibacterial and anti-biofilm testing as the pure compounds. The relative concentrations of CBD and THC in the resulting oils were not known or determined and information not provided by the supplier.

Table S1. A systematic review on plant-based natural compounds (PBCs) as an antibacterial agent.

Study ID	Pub	PBCs	Bacteria evaluated	MIC (μg/ml)	Comment/s	Ref	Quality Score
Esra	2020	*Cannabis sativa* extracts (Seeds oil)	*S. aureus*	**25**	The oil of the seeds of *Cannabis sativa* exerted pronounced antibacterial activity (21 - 28 mm) against *Bacillus subtilis* and *Staphylococcus aureus*, moderate activity (15 mm) against *Escherichia coli* and high activity (16 mm) against *Pseudomonas aeruginosa* and inactive against the two fungi tested.	(24)	7
			E. coli	**25**			
			P. aeruginosa	**50**			
		Cannabis sativa extracts (Whole plant oil)	*S. aureus*	**50**	The petroleum ether extract of the whole plant exhibited pronounced antibacterial activity (23 - 28 mm) against both *Bacillus subtilis* and *Staphylococcus aureus* organisms, high activity (16 mm) against *Escherichia coli* and inactive against *Pseudomonas aeruginosa* and both fungi.		
			E. coli	**25**			
			P. aeruginosa	**125**			
Appendino	2008	Cannabinoids and their analogues	13# Drug-Resistant Strains of *S. aureus*	**4 (1-128)**	methylation and acetylation of the phenolic hydroxyls, esterification of the carboxylic group of pre-cannabinoids, and introduction of a second prenyl moiety were all detrimental for antibacterial activity.	(25)	8
Radwan	2009	9 Cannabinoids isolated from a high-potency variety of Cannabis sativa	MRSA	**7-53**	Compounds 6 and 7 displayed significant antibacterial and antifungal activities, respectively, while 5 displayed strong antileishmanial activity. Strong antileishmanial activity Significant antibacterial and antifungal activities	(26)	7
			S. aureus	**3-30**			
			E. coli	**54**			
			M. intracellulare	**30**			
Nissen	2010	freshly extracted essential oils from three legal (THCb0.2% w/v) hemp varieties (Carmagnola, Fibranova and Futura)	Gram (+), opportunistic and moderate pathogenic bacteria including Clostridium spp. and Enterococcus spp.;	**>2-11 (% v/v)**	essential oils of industrial hemp can significantly inhibit microbial growth, to an extent depending on variety and sowing time.<br>Resulted in effective control of *Enterococcus hirae, Enterococcus faecium and S. salivarius* subsp. thermophilus.	(27)	7
			Gram (−), phytopathogens bacteria including Pseudomonas spp. and Pectobacterium spp.;	**>2-1.05 (% v/v)**	Futura confirmed the best results even on Gram (−), with MIC values always well below the threshold limit (2.00% v/v). both Carmagnola and Fibranova-II were above the MIC limit only in one case out of seven: *Pseudomonas savastonoi and Pectobacterium carotovorum* subsp. carotovorum, respectively for the two varieties.<br>alpha-pinene was again the most effective compound for contrasting Gram (−) bacteria		
Sarmadyan	2014	hydro-alcoholic extract of cannabis	*E. coli*	**50**	The maximum anti-microbial effect of the hydro-alcoholic extract of cannabis was seen for gram-positive cocci, especially *S. aureus*, whereas nonfermentative gram negatives presented resistance to the extract. This extract had an intermediate effect on Enterobacteriacae family. Cannabis components extracted through chemical analysis can perhaps be effective in the treatment of nosocomial infections	(28)	6
			E. coli ESBL +	**100**			
			S. aureus	**25**			
			MRSA	**50**			
			P. aeruginosa ESBL+	**>100**			
			P. aeruginosa	**100**			
			K. pneumoniae	**100**			
			A. baumannii	**>100**			
Thu Vu	2015	Methanol extracts *Cannabis sativa* (Cannabis sativa)	*B. cereus*	**2000**	Gram (−) bacteria were less susceptible to *Cannabis sativa*	(29)	7
			S. aureus	**2000**			
			E. coli	**>2000**			
			P. aeruginosa	**>2000**			
Lelario	2018	Hemp-type C. sativa extract	*B. cereus*	**5**	Hemp-type *C. sativa* extract showed antimicrobial activity only against Gram+ bacteria, but the main individual components tested showed always a limited bioactivity	(30)	7
			B. thuringiensis	**5-10**			
			B. amyloliquefaciens	**5**			
			P. orientalis	**NI**			
			P. orientalis	**NI**			
Zengin	2018	hemp EO, parts of C. sativa	*S. aureus*, ATCC and 3 clinical isoaltes	**8000**	the antibacterial and antibiofilm activities of hemp EO suggested it could be a possible candidate for the treatment of infections related to those abovementioned microorganisms	(31)	8

Iseppi	2019	17 hemp EOs	*S. aureus* ATCC and food samples	**2-16**	Seventeen essential oils from different fibre-type varieties of *C. sativa* (industrial hemp or hemp) using GC-MS and GC-FID techniques.<br>The results showed good antibacterial activity of six hemp essential oils against the Gram-positive bacteria, thus suggesting that hemp essential oil can inhibit or reduce bacterial proliferation and can be a valid support to reduce microorganism contamination, especially in the food processing field.	(32)	8
		Ciprofloxacin	*S. aureus* ATCC and food samples	**0.5-16**			
		17 hemp EOs	*S. epidermidis* food sample	**1-16**			
		Ciprofloxacin	*S. epidermidis* food sample	**0.5**			
		17 hemp EOs	*L. monocytogenes* ATCC and food samples	**1-16**			
		17 hemp EOs	*E. faecalis* ATCC and food samples	**0.5-32**			
		Ciprofloxacin	*E. faecalis* ATCC and food samples	**0.5-16**			
		17 hemp EOs	*E. hirae* ATCC and food samples	**4-32**			
		Ciprofloxacin	*E. hirae* ATCC and food samples	**8**			
		17 hemp EOs	*E. faecium* ATCC	**1-16**			
		Ciprofloxacin	*E. faecium* ATCC	**4-8**			
		17 hemp EOs	*B. subtilis* ATCC	**2-16**			
		17 hemp EOs	*B. cereus* EB 362	**2 (1-16)**			
		CDB	*S. aureus* ATCC and food samples	**8-32**			
		CDB	*S. epidermidis* food sample	**16**			
		CDB	*L. monocytogenes* ATCC and food samples	**1-4**			
		CDB	*E. faecalis* ATCC and food samples	**1-4**			
		CDB	*E. faecium* ATCC	**1-4**			
		CDB	*E. hirae* ATCC and food samples	**2**			
		CDB	*B. subtilis* ATCC	**8**			
		CDB	*B. cereus* EB 362	**8**			
Palmieri	2021	10# *Cannabis sativa* essential oils	*L. monocytogenes* ATCC	**>20 (0.625- >20)**	except for Futura 75, the effect of time on the antimicrobial activity was variable and requires further investigations; nevertheless, the inhibitory activity of all EOs against *Pseudomonas fluorescens* P34 was significant.	(33)	8
			S. aureus	**>20 (0.15- >20)**			
			P. fluorescens P34	**1.5 (0.31-2-5)**			
			B. thermosphacta B1	**2.5 (0.31->20)**			
			S. Enteriditis S2	**>20 (10- >20)**			
			S. Typhimurium S4	**>20**			
			E. faecium ATCC	**2.5 (0.625 - >20)**			
Claudia	2021	two extracts from a new Chinese accession of *Cannabis sativa* L. (Δ9 - tetrahydrocannabinol <0.2%)	*S. aureus* ATCC	**39**	two extracts from a new Chinese accession (G-309) of *Cannabis sativa* L. (Δ9 - tetrahydrocannabinol <0.2%)	(34)	7
			19# MRSA clinical strains	**39**			
Blaskovich	2021	CBD	*S. aureus*, MRSA	**1-2**	results demonstrate that cannabidiol has excellent activity against biofilms, little propensity to induce resistance, and topical in vivo efficacy.<br>selectively kill a subset of Gram-negative bacteria that includes the ‘urgent threat’ pathogen *N. gonorrhoeae.*<br>CBD does not lead to resistance after repeated exposure.	(35)	9
			S. epidermidis	**1-2**			
			S. pneumoniae	**1-4**			
			S. pyogenes	**1**			
			E. faecium	**0.5-1**			
			E. faecalis	**2-4**			
			C. difficile	**2-4**			
			C. acnes	**1-2**			
			Gram (-) such as the key ESKAPE pathogens *E. coli, K. pneumoniae, P. aeruginosa* and *A. baumannii*	**>64**			
			N. gonorrhoeae	**1**			
			N. meningitidis	**0.25**			
			L. pneumophila	**1**			
Martinenghi	2020	CBDA	S. aureus ATCC, MRSA	**2, 4**	Two compounds were extracted by ethanol, purified on a C18 sep-pack column.	(36)	8
		CBDA	*S. epidermidis*	**4**			

		CBDA	*E. coli*	**>64**	CBD displayed a substantial inhibitory effect on Gram-positive bacteria with minimal inhibitory concentrations ranging from 1 to 2 µg/mL CBDA presented a two-fold lower antimicrobial activity than its decarboxylated form		
		CBDA	*P. aeruginosa*	**>64**			
		CBD	*S. aureus* ATCC, MRSA	**1**			
		CBD	*S. epidermidis*	**2**			
		CBD	*E. coli*	**>64**			
		CBD	*P. aeruginosa*	**>64**			
Paulo, Valle, Ingmer, and Zetterström, Martínez, Sun, Makobongo	2010, 2016, 2019, 2013, 2020, 2012, 2014	Resveratrol	*E. coli* EHEC	**10**	Resveratrol has antibacterial activity against all tested Gram-positive bacteria using both the disk diffusion and broth microdilution methods.	(37)	8, 7, 8, 7, 7, 7, 8
			B. cereus ATCC	**50**		(37)	
			S. aureus ATCC	**100**		(37)	
			3# MRSA and MSSA	**200 (100-200)**		(37)	
			E. faecalis ATCC	**100-1000**		(37-39)	
			Gram (-) including *E.coli, K. pneumoniae, S. typhimurium,* and *S. aeruginosa*	**>400**		(37, 39, 40)	
			E. faecium D344R	**128**		(41)	
			M. tuberculosis H37Rv	**100**		(42)	
			S. pyogenes	**>200**		(39)	
			B. cereus ATCC, NCTR	**50, 1000**		(39)	
			S. aureus	**100- >1000**		(39)	
			M. tuberculosis H37Rv	**100**		(39)	
			S. pneumoniae HM145	**100**		(39)	
			H. pylori	**25-100**		(40, 43)	
Lemos, Imelouane, Ahmad, Fournomiti	2017, 2009, 2014, 2015	Thyme Essential oils	*S. aureus*	**20-1000**	The period of the harvests affected the chemical composition, antioxidant activity, and antimicrobial activity of thyme essential oils, and these differences can be related to seasonal variations of temperature and humidity. The largest antioxidant and antimicrobial activities were displayed by the essential oil produced in spring (October/2012).	(44-46)	7, 7, 8, 8
			E. coli	**500-128000**		(44-47)	
			S. typhimurium	**500-750**		(44)	
			S. epidermidis ATCC	**1330**		(45)	
			Streptococcus sp	**2670**		(45)	
			M. cattarhalis ATCC	**1000**		(46)	
			St. aureus ATCC	**500**		(46)	
			B. cereus ATCC	**500**		(46)	
			K. pneumoniae	**16000-32000**		(47)	
Imelouane, Veldhuizen, Guarda, Javier, Chueca, AL-Ani, Cacciatore, Mariri, Cacciatore	2009, 2006, 2011, 2019, 2016, 2015, 2015, 2015	Carvacrol	*S. aureus*	**250-1700, 250 ppm**	CAR interacts with the lipid bilayer of the bacterial cytoplasmic membrane due to its hydrophobic nature and aligns itself between fatty acid chains causing the expansion and destabilization of the membrane structure by increasing its fluidity and permeability for protons and ions. The loss of the ion gradient leads to bacterial cell death	(44, 48-51)	7, 7, 7, 7, 8, 7, 7, 8
			E. coli	**250-1200, 250 ppm**		(48) (44, 49, 51-54)	
			S. typhimurium	**<0.4- 375**		(44, 51, 54, 55)	
			K. pneumoniae ATCC,	**3-300**		(51, 54)	
			S. enteritidis	**187**		(55)	
			Y. enterocolitica O9	**0.75**		(51, 54)	
			P. aeruginosa	**6-500**		(51, 53, 54)	
			Proteus spp	**<0.375**		(54)	
Yu-Meng Song,	2020	Tea tree oil	*S. mutans*	**0.125%**	0.25% (MBC)	(56)	8
Shi	2018		*E. coli*	**2 (2-4)**		(57),	9
Shi	2018		*L. monocytogenes*	**1 (1-2)**		(57),	
Brun	2019	10 # Tea tree oil	MRSA	**0.5-2.5 (%v/v)**		(58)	8
Brun	2019		*P. aeruginosa*	**0.25-2 (%v/v)**		(58)	
Karpanen	2008	Tea tree oil	*S. epidermidis*	**2000-16000**	there may be a role for essential oils, in particular EO, for improved skin antisepsis when combined with chlorhexidine digluconate	(59)	7
Low,	2011		*S. aureus*	**0.5-2 (%v/v)**		(60)	7
			P. aeruginosa	**1-16 (%v/v)**			
Firmino	2018	Cinnamaldehyde	*S. aureus*	**250**	In cell viability tests, 2 mg/ml of cinnamaldehyde reduced the number of viable cells by 5.74 Log CFU/ml.	(61)	8
			E. coli	**250**			
			S. epidermidis	**250**			
			S. pyogenes	**500**			
			P. aeruginosa	**500**			
Khan	2005		*H. pylori*	**2**		(62)	8
Zhiya	2019		*S. mutans*	**1000**	cinnamaldehyde at sub-MIC level suppressed the microbial activity on *S. mutans* biofilm by modulating hydrophobicity, aggregation, acid production, acid tolerance, and virulence gene expression.	(63)	7

Krist	2014	cis-Nerolidol, rans-Nerolidol	*E.coli* ATCC	**0.2**		(64)	8
			P.aeruginosa	**0.4**			
			S. aureus	**0.1**			
			S.epidermidis	**0.1**			
			B.cereus ATCC	**0.1**			
Khatkar, Jorge, Doria	2017, 2008, 2019	36# p-coumaric acid	*S. aureus*	**1.67->2000**		(65, 66)	7,8
			B. subtilis	**2**		(65)	7
			E. coli	**1.7->300**		(65-67)	7,8, 8
Jorge, Doria	2008, 2019	p-coumaric acids	*P. aeruginosa*	**>2000**	All the evaluated propolis samples exhibited similar antibacterial activity, but different contents of prenylated p-coumaric acids throughout the year.	(66, 67)	8, 8
Doria	2019		*S. epidermidis*	**<15->2000**		(66)	8
	2019		*A. baumannii*	**250->2000**		(66)	8
Mandroli	2013	Curcumin	*S. mutans*	**333.3**		(68)	7
			L. casei	**125**			
			L. casei	**167.67**			
			P. gingivalis	**125**			
			P. intermedia	**208.33**			
Izui	2016		*A.actinomycetemcomitans*	**> 100**	Curcumin possesses antibacterial activity against periodontopathic bacteria and may be a potent agent for preventing periodontal diseases	(69)	8
			F. nucleatum	**10**			
			P. gingivalis	**15**			

Essential oils (EOs), cannabidiol acid (CBDA), cannabidiol (CBD).
Joanna Briggs Institute checklist questions for quality assessment of included studies are as follows.

Q1= Was the sample frame appropriate to address the target population?
Q2= Were study participants sampled in an appropriate way?
Q3= Was the sample size adequate?
Q4= Were the study subjects and the setting described in detail?
Q5= Was the data analysis conducted with sufficient coverage of the identified sample?
Q6= Were valid methods used for the identification of the condition?
Q7= Was the condition measured in a standard, reliable way for all participants?
Q8= Was there appropriate statistical analysis?
Q9= Was the response rate adequate, and if not, was the low response rate managed appropriately?

Table S2. Minimum inhibitory concentration (MIC), minimum bactericidal concentration (MBC), and minimum biofilm inhibitory concentration (MBIC) of nine PBCs against *P. aeruginosa*, *S. aureus*, *E. coli*, *K. pneumoniae*, *P. mirabilis*, and *A. baumannii.*

	**Bacteria**	**Genta micin**	**Cipro floxacin**	**Canna bidiol**	**THC**	**Cinnamal dehyde**	**Tea Tree Oil**	**Nerolidol**	**Carvacrol**	***0*-coumaric acid**	**Thymol**	**Canada Balsam**
**MIC (mM)**	*E.coli*	0.0039	0.0001	0.04	0.72	0.37	3.08	15.63	0.24	125.00	0.80	16.07
	K. pneumonia	0.0006	0.0004	0.40	0.72	1.46	6.15	312.50	0.98	156.25	0.20	13.39
	P. aeruginosa	0.0023	0.0008	0.20	0.18	3.91	29.53	375.00	15.63	250.00	25.00	8.04
	P. miriablis	0.0023	0.0001	0.80	0.72	1.22	5.23	375.00	1.10	70.31	0.20	21.43
	A. baumannii	1.60	0.40	0.35	**0.40**	**0.03**	8.00	250.00	**0.0015**	125.00	**0.06**	6.25
	S. aureus	0.0063	0.0016	**0.0002**	0.18	3.91	4.92	250.00	0.98	125.00	15.60	1.34
**MBC (mM)**	*E.coli*	0.0070	0.0001	0.04	0.36	1.95	2.61	500.00	0.24	78.13	0.80	21.43
	K. pneumonia	0.0016	0.0004	1.60	0.14	2.44	14.77	125.00	0.98	187.50	0.20	10.71
	P. aeruginosa	0.0047	0.0016	0.40	0.36	7.81	39.38	500	140.63	250.00	25.00	10.71
	P. miriablis	0.0023	0.0001	0.80	0.72	1.22	5.54	500	1.10	19.53	0.40	16.07
	A. baumannii	1.80	0.40	3.20	2.90	**0.20**	16.00	500	**0.03**	250.00	**0.13**	250.00
	S. aureus	0.0033	0.0039	0.40	0.18	4.03	2.61	7.81	0.61	140.63	15.60	0.68
**MBIC (mM)**	*E.coli*	0.0006	0.0001	0.80	0.72	0.37	5.08	7.81	0.24	11.72	0.20	NA
	K. pneumonia	0.0008	0.0004	1.60	0.72	1.46	14.77	281.25	0.98	140.63	0.40	NA
	P. aeruginosa	0.0023	0.0012	0.80	0.36	5.86	29.53	500	23.44	250.00	25.00	NA
	P. miriablis	0.0023	0.0001	0.40	0.72	1.22	5.23	500	1.10	62.50	0.20	NA
	A. baumannii	1.60	0.1000	0.80	0.70	**0.03**	8.00	250	**0.003**	125.00	**0.06**	62.50
	S. aureus	AN	AN	0.80	NA	NA	NA	NA	NA	NA	NA	NA

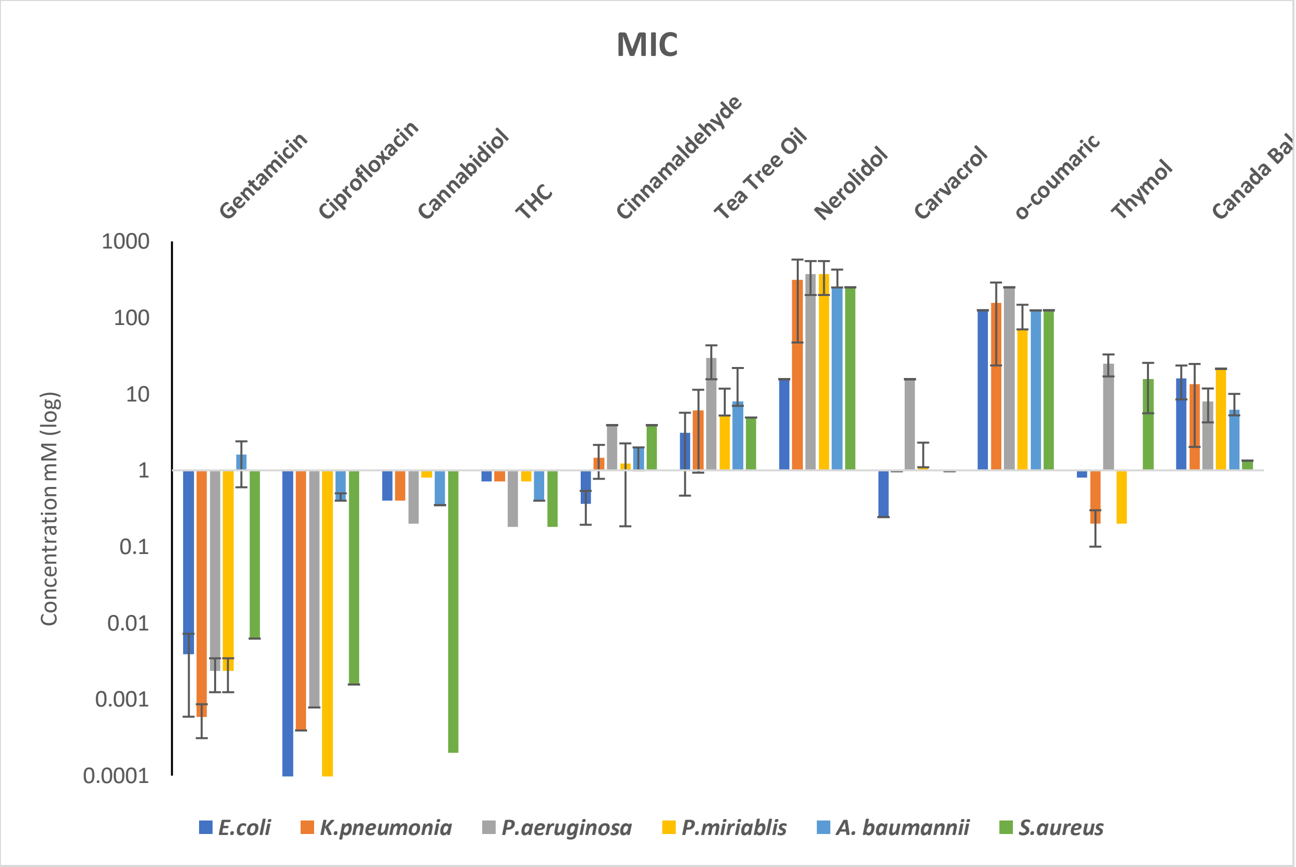

Figure S1. Bacteriostatic potency of natural products in comparison with gentamycin and ciprofloxacin (n=3).

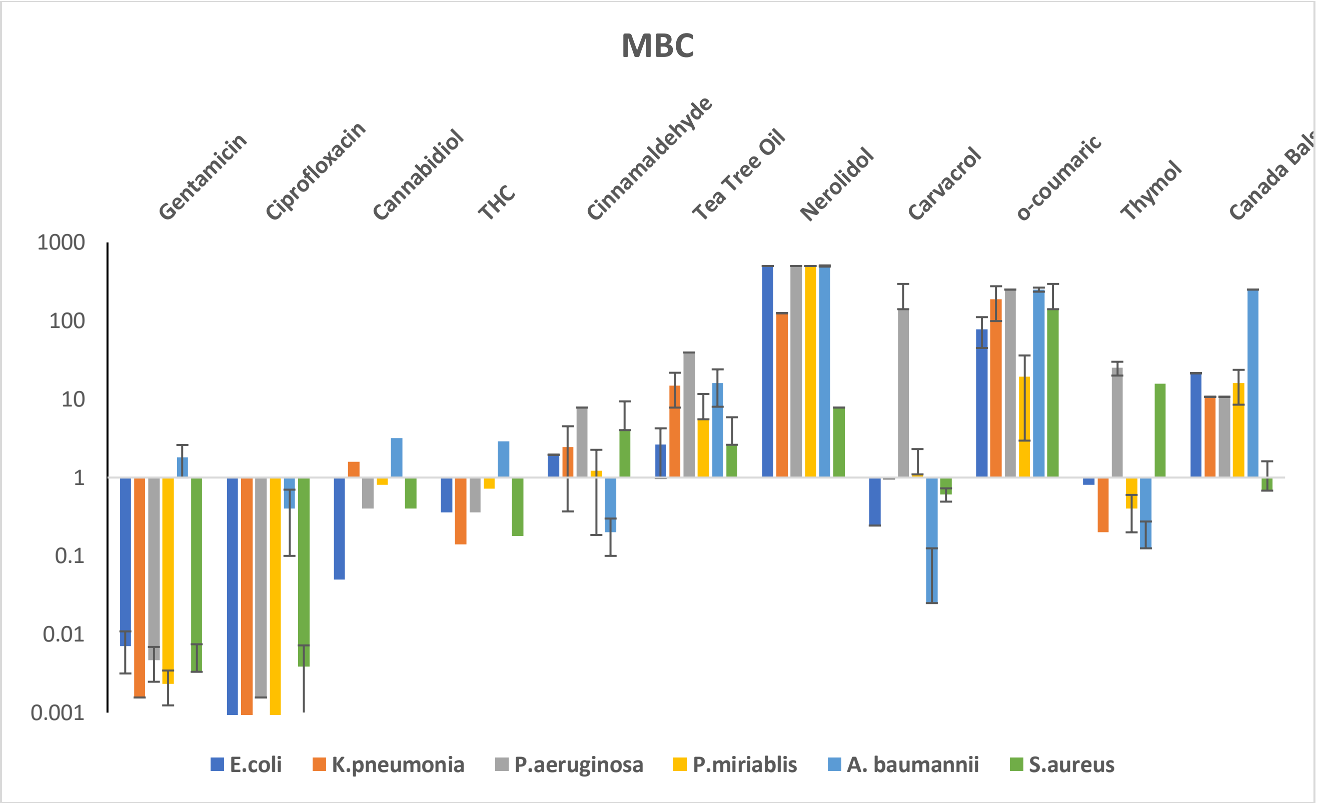

Figure S2. Bactericidal potency of natural products in comparison with gentamycin and ciprofloxacin (n=3).

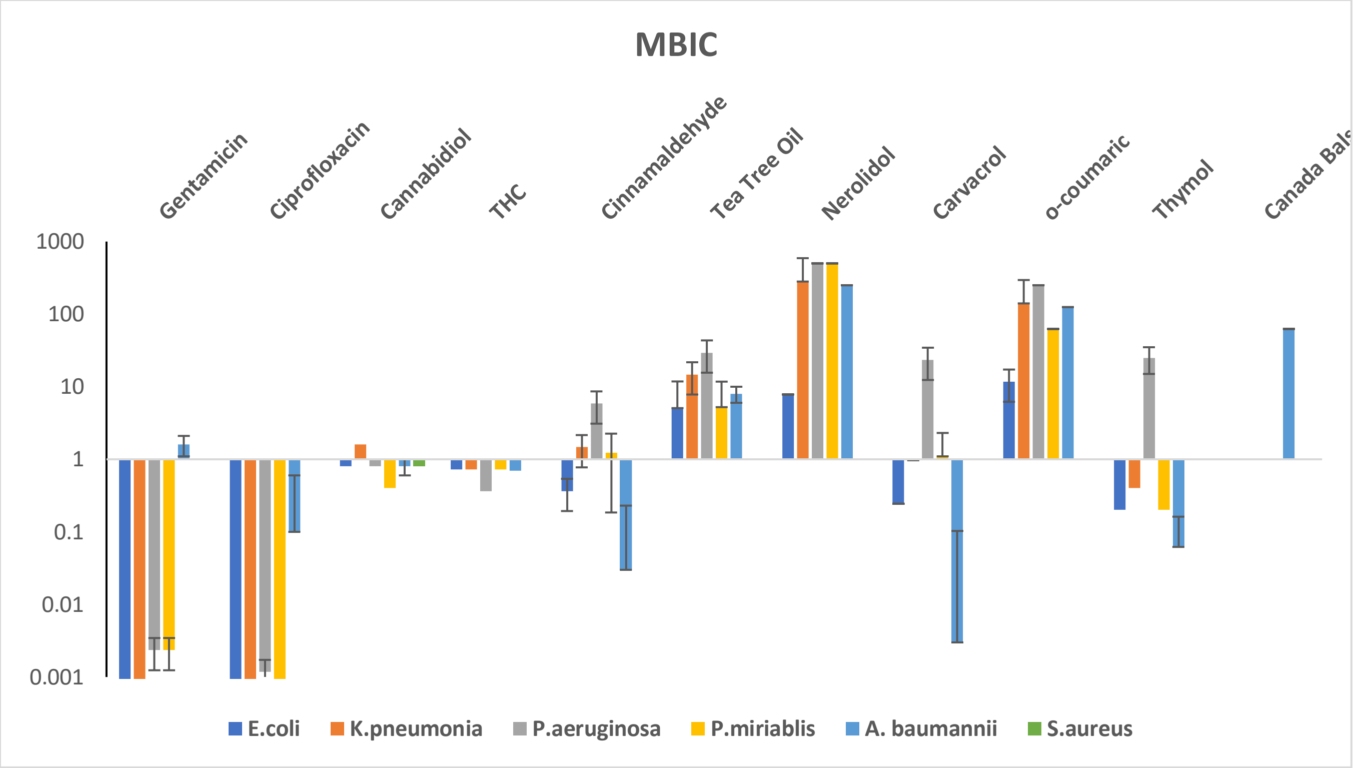

Figure S3. Biofilm inhibition potency of natural products in comparison with gentamycin and ciprofloxacin (n=3).

Refences

1. Moher D, Liberati A, Tetzlaff J, Altman DG, & Group P (2009) Preferred reporting items for systematic reviews and meta-analyses: the PRISMA statement. *PLoS medicine* 6(7):e1000097.
2. Santos WMd, Secoli SR, & Püschel VAdA (2018) The Joanna Briggs Institute approach for systematic reviews. *Revista latino-americana de enfermagem* 26.
3. https://wwwn.cdc.gov/ARIsolateBank/Panel/IsolateDetail?IsolateID=33
4. Lemire JA, Kalan L, Bradu A, & Turner RJ (2015) Silver oxynitrate, an unexplored silver compound with antimicrobial and antibiofilm activity. *Antimicrobial agents and chemotherapy* 59(7):4031-4039.
5. Monych NK & Turner RJ (2020) Multiple Compounds Secreted by Pseudomonas aeruginosa Increase the Tolerance of Staphylococcus aureus to the Antimicrobial Metals Copper and Silver. *mSystems* 5(5).
6. Agarwal G, Kapil A, Kabra S, Das BK, & Dwivedi S (2005) In vitro efficacy of ciprofloxacin and gentamicin against a biofilm of Pseudomonas aeruginosa and its free-living forms. *National Medical Journal of India* 18(4):184.
7. Ceri H*, et al.* (1999) The Calgary Biofilm Device: new technology for rapid determination of antibiotic susceptibilities of bacterial biofilms. *Journal of clinical microbiology* 37(6):1771-1776.

8. Diepoltová A, Konečná K, Janďourek O, & Nachtigal P (2021) Study of the impact of cultivation conditions and peg surface modification on the in vitro biofilm formation of Staphylococcus aureus and Staphylococcus epidermidis in a system analogous to the Calgary biofilm device. *Journal of Medical Microbiology* 70(5):001371.
9. O'Toole GA (2011) Microtiter dish biofilm formation assay. *JoVE (Journal of Visualized Experiments)* (47):e2437.
10. https://assets.thermofisher.com/TFS-Assets/LSG/manuals/mp36003.pdf
11. https://www.thermofisher.com/document-connect/document-connect.html?url=https%3A%2F%2Fassets.thermofisher.com%2FTFS-Assets%2FLSG%2Fmanuals%2FMAN0011275_Pierce_Quant_Peroxide_Asy_UG.pdf (
12. Hennessy DJ, Reid GR, Smith FE, & Thompson SL (1984) Ferene—a new spectrophotometric reagent for iron. *Canadian journal of chemistry* 62(4):721-724.
13. Morones-Ramirez JR, Winkler JA, Spina CS, & Collins JJ (2013) Silver enhances antibiotic activity against gram-negative bacteria. *Science translational medicine* 5(190):190ra181-190ra181.
14. Novo DJ, Perlmutter NG, Hunt RH, & Shapiro HM (2000) Multiparameter flow cytometric analysis of antibiotic effects on membrane potential, membrane permeability, and bacterial counts of Staphylococcus aureus and Micrococcus luteus. *Antimicrobial agents and chemotherapy* 44(4):827-834.
15. LeBel M (1988) Ciprofloxacin: chemistry, mechanism of action, resistance, antimicrobial spectrum, pharmacokinetics, clinical trials, and adverse reactions. *Pharmacotherapy: The Journal of Human Pharmacology and Drug Therapy* 8(1):3-30.
16. TANGY F, MOUKKADEM M, VINDIMIAN E, CAPMAU ML, & LE GOFFIC F (1985) Mechanism of action of gentamicin components: Characteristics of their binding to Escherichia coli ribosomes. *European journal of biochemistry* 147(2):381-386.
17. Jung HJ & Lee DG (2008) Synergistic antibacterial effect between silybin and N, N'-dicyclohexylcarbodiimide in clinical Pseudomonas aeruginosa isolates. *The Journal of Microbiology* 46(4):462-467.
18. Linnett P & Beechey R (1979) [59] Inhibitors of the ATP synthetase systems. *Methods in enzymology* 55:472-518.
19. Brenner S (1974) The genetics of Caenorhabditis elegans. *Genetics* 77(1):71-94.
20. Stiernagle T (2006) Maintenance of C. elegans (February 11, 2006), WormBook, ed. The C. elegans Research Community, WormBook, doi/10.1895/wormbook. 1.101. 1.
21. Rahman I & MacNee W (2000) Oxidative stress and regulation of glutathione in lung inflammation. *European Respiratory Journal* 16(3):534-554.
22. Arulselvan P*, et al.* (2016) Role of antioxidants and natural products in inflammation. *Oxidative medicine and cellular longevity* 2016.
23. Gill R, Tsung A, & Billiar T (2010) Linking oxidative stress to inflammation: Toll-like receptors. *Free Radical Biology and Medicine* 48(9):1121-1132.
24. Karas JA*, et al.* (2020) The antimicrobial activity of cannabinoids. *Antibiotics* 9(7):406.
25. Appendino G*, et al.* (2008) Antibacterial cannabinoids from Cannabis sativa: a structure− activity study. *Journal of natural products* 71(8):1427-1430.
26. Radwan MM*, et al.* (2009) Biologically active cannabinoids from high-potency Cannabis sativa. *Journal of natural products* 72(5):906-911.
27. Nissen L*, et al.* (2010) Characterization and antimicrobial activity of essential oils of industrial hemp varieties (Cannabis sativa L.). *Fitoterapia* 81(5):413-419.
28. Sarmadyan H, Solhi H, Najarian-Araghi N, & Ghaznavi-Rad E (2014) Determination of the Antimicrobial Effects of Hydro-Alcoholic Extract of Cannabis Sativa on Multiple Drug Resistant Bacteria Isolated from Nosocomial Infections. *Iranian Journal of Toxicology* 7(23):967-972.

29. Vu TT, *et al.* (2015) In vitro antibacterial activity of selected medicinal plants traditionally used in Vietnam against human pathogenic bacteria. *BMC Complementary and Alternative Medicine* 16(1):1-6.
30. Lelario F, *et al.* (2018) Identification and antimicrobial activity of most representative secondary metabolites from different plant species. *Chemical and Biological Technologies in Agriculture* 5(1):1-12.
31. Zengin G, *et al.* (2018) Chromatographic analyses, in vitro biological activities, and cytotoxicity of Cannabis sativa L. essential oil: A multidisciplinary study. *Molecules* 23(12):3266.
32. Iseppi R, *et al.* (2019) Chemical characterization and evaluation of the antibacterial activity of essential oils from fibre-type Cannabis sativa L.(Hemp). *Molecules* 24(12):2302.
33. Palmieri S, *et al.* (2021) Effect of the Distillation Time on the Chemical Composition, Antioxidant Potential and Antimicrobial Activity of Essential Oils from Different Cannabis sativa L. Cultivars. *Molecules* 26(16):4770.
34. Muscarà C, *et al.* (2021) Antioxidant and antimicrobial activity of two standardized extracts from a new Chinese accession of non-psychotropic Cannabis sativa L. *Phytotherapy Research* 35(2):1099-1112.
35. Blaskovich MA, *et al.* (2021) The antimicrobial potential of cannabidiol. *Communications Biology* 4(1):1-18.
36. Martinenghi LD, Jønsson R, Lund T, & Jenssen H (2020) Isolation, Purification, and antimicrobial characterization of cannabidiolic acid and cannabidiol from Cannabis sativa L. *Biomolecules* 10(6):900.
37. Paulo L, Ferreira S, Gallardo E, Queiroz JA, & Domingues F (2010) Antimicrobial activity and effects of resveratrol on human pathogenic bacteria. *World Journal of Microbiology and Biotechnology* 26(8):1533-1538.
38. del Valle P, *et al.* (2016) Antimicrobial activity of kaempferol and resveratrol in binary combinations with parabens or propyl gallate against Enterococcus faecalis. *Food Control* 61:213-220.
39. Vestergaard M & Ingmer H (2019) Antibacterial and antifungal properties of resveratrol. *International Journal of Antimicrobial Agents* 53(6):716-723.
40. Zetterström CE, *et al.* (2013) The resveratrol tetramer (-)-hopeaphenol inhibits type III secretion in the gram-negative pathogens Yersinia pseudotuberculosis and Pseudomonas aeruginosa. *PLoS One* 8(12):e81969.
41. Álvarez-Martínez FJ, Barrajón-Catalán E, Encinar JA, Rodríguez-Díaz JC, & Micol V (2020) Antimicrobial capacity of plant polyphenols against gram-positive bacteria: A comprehensive review. *Current medicinal chemistry* 27(15):2576-2606.
42. Sun D, *et al.* (2012) Evaluation of flavonoid and resveratrol chemical libraries reveals abyssinone II as a promising antibacterial lead. *ChemMedChem* 7(9):1541.
43. Makobongo MO, Gilbreath JJ, & Merrell DS (2014) Nontraditional therapies to treat Helicobacter pylori infection. *Journal of Microbiology* 52(4):259-272.
44. Lemos MF, *et al.* (2017) Seasonal variation affects the composition and antibacterial and antioxidant activities of Thymus vulgaris. *Industrial Crops and Products* 95:543-548.
45. Imelouane B, *et al.* (2009) Chemical composition and antimicrobial activity of essential oil of thyme (Thymus vulgaris) from Eastern Morocco. *Int. J. Agric. Biol* 11(2):205-208.
46. Ahmad A, Van Vuuren S, & Viljoen A (2014) Unravelling the complex antimicrobial interactions of essential oils—the case of Thymus vulgaris (Thyme). *Molecules* 19(3):2896-2910.
47. Fournomiti M, *et al.* (2015) Antimicrobial activity of essential oils of cultivated oregano (Origanum vulgare), sage (Salvia officinalis), and thyme (Thymus vulgaris) against clinical isolates

of Escherichia coli, Klebsiella oxytoca, and Klebsiella pneumoniae. *Microbial ecology in health and disease* 26(1):23289.
48. Veldhuizen EJ, Tjeerdsma-van Bokhoven JL, Zweijtzer C, Burt SA, & Haagsman HP (2006) Structural requirements for the antimicrobial activity of carvacrol. *Journal of agricultural and Food Chemistry* 54(5):1874-1879.
49. Guarda A, Rubilar JF, Miltz J, & Galotto MJ (2011) The antimicrobial activity of microencapsulated thymol and carvacrol. *International journal of food microbiology* 146(2):144-150.
50. Rúa J, Del Valle P, de Arriaga D, Fernández-Álvarez L, & García-Armesto MR (2019) Combination of carvacrol and thymol: Antimicrobial activity against Staphylococcus aureus and antioxidant activity. *Foodborne pathogens and disease* 16(9):622-629.
51. Issam A-A, Zimmermann S, Reichling J, & Wink M (2015) Pharmacological synergism of bee venom and melittin with antibiotics and plant secondary metabolites against multi-drug resistant microbial pathogens. *Phytomedicine* 22(2):245-255.
52. Chueca B, Berdejo D, Gomes-Neto NJ, Pagan R, & Garcia-Gonzalo D (2016) Emergence of hyper-resistant Escherichia coli MG1655 derivative strains after applying sub-inhibitory doses of individual constituents of essential oils. *Frontiers in microbiology* 7:273.
53. Cacciatore I*, et al.* (2015) Carvacrol codrugs: a new approach in the antimicrobial plan. *PLoS One* 10(4):e0120937.
54. Al-Mariri A, Swied G, Oda A, & Al Hallab L (2013) Antibacterial activity of thymus syriacus boiss essential oil and its components against some Syrian gram-negative bacteria isolates. *Iranian journal of medical sciences* 38(2 Suppl):180.
55. Du E*, et al.* (2015) In vitro antibacterial activity of thymol and carvacrol and their effects on broiler chickens challenged with Clostridium perfringens. *Journal of animal science and biotechnology* 6(1):1-12.
56. Song Y-M*, et al.* (2020) In vitro evaluation of the antibacterial properties of tea tree oil on planktonic and biofilm-forming Streptococcus mutans. *AAPS PharmSciTech* 21(6):1-12.
57. Shi C, Zhang X, & Guo N (2018) The antimicrobial activities and action-mechanism of tea tree oil against food-borne bacteria in fresh cucumber juice. *Microbial pathogenesis* 125:262-271.
58. Brun P, Bernabè G, Filippini R, & Piovan A (2019) In vitro antimicrobial activities of commercially available tea tree (Melaleuca alternifolia) essential oils. *Current microbiology* 76(1):108-116.
59. Karpanen TJ, Worthington T, Hendry E, Conway BR, & Lambert PA (2008) Antimicrobial efficacy of chlorhexidine digluconate alone and in combination with eucalyptus oil, tea tree oil and thymol against planktonic and biofilm cultures of Staphylococcus epidermidis. *Journal of Antimicrobial Chemotherapy* 62(5):1031-1036.
60. Low W, Martin C, Hill D, & Kenward M (2011) Antimicrobial efficacy of silver ions in combination with tea tree oil against Pseudomonas aeruginosa, Staphylococcus aureus and Candida albicans. *International journal of antimicrobial agents* 37(2):162-165.
61. Firmino DF*, et al.* (2018) Antibacterial and antibiofilm activities of Cinnamomum sp. essential oil and cinnamaldehyde: antimicrobial activities. *The Scientific World Journal* 2018.
62. Ali SM*, et al.* (2005) Antimicrobial activities of Eugenol and Cinnamaldehyde against the human gastric pathogen Helicobacter pylori. *Annals of clinical microbiology and antimicrobials* 4(1):1-7.
63. He Z, Huang Z, Jiang W, & Zhou W (2019) Antimicrobial activity of cinnamaldehyde on Streptococcus mutans biofilms. *Frontiers in microbiology* 10:2241.
64. Krist S*, et al.* (2015) Antimicrobial activity of nerolidol and its derivatives against airborne microbes and further biological activities. *Natural product communications* 10(1):1934578X1501000133.

65. Khatkar A, Nanda A, Kumar P, & Narasimhan B (2017) Synthesis, antimicrobial evaluation and QSAR studies of p-coumaric acid derivatives. *Arabian Journal of Chemistry* 10:S3804-S3815.
66. Forero-Doria O, Araya-Maturana R, Barrientos-Retamal A, Morales-Quintana L, & Guzmán L (2019) N-alkylimidazolium salts functionalized with p-coumaric and cinnamic acid: a study of their antimicrobial and antibiofilm effects. *Molecules* 24(19):3484.
67. Jorge R*, et al.* (2008) Brazilian propolis: seasonal variation of the prenylated p-coumaric acids and antimicrobial activity. *Pharmaceutical Biology* 46(12):889-893.
68. Mandroli PS & Bhat K (2013) An in-vitro evaluation of antibacterial activity of curcumin against common endodontic bacteria. *Journal of Applied Pharmaceutical Science* 3(10):16.
69. Izui S*, et al.* (2016) Antibacterial activity of curcumin against periodontopathic bacteria. *Journal of periodontology* 87(1):83-90.
